# Hand Pressure and Soft Foil, vs. Malleting and Cohesive Foil

**Published:** 1878-01

**Authors:** J. Foster Flagg


### ARTICLE VI.

### *Hand Pressure and Soft Foil, vs. Malleting and Cohesive Foil.*

BY J. FOSTER FLAGG, D. D. S.

Recognizing that gold foil is yet the most acceptable material for filling a very large class of cavities of decay in a large proportion of teeth, I desire to place myself upon the side of the utmost possible return to the use of hand-pressure and soft foil, urging this practice from the standpoints of *economy of time*, both to patient and operator, and *durability of filling*, as shown in my experience by the tabulated statistics of fifteen years.

It is now a sufficient length of time since I had wrought with cohesive gold and mallets of various kinds (wooden, leaden and steel,) to have attained that degree of proficiency which, in my estimation warranted a commencement of comparison as to ultimate results.

Since then, and during my experiments, I have used in addition to the hard mallets, several of the automatics (preferring the Snow & Lewis,) the engine mallet, and the "Electric." My experience with the last two forms of mallet has been too limited to be of any value to others besides myself, and their acceptable construction is of too recent date to admit of any reasonably lengthy test for durability of work.

My reason for advocating the practice of hand-pressure and the use of soft foil is the fact that my mallet work of ten years, standing does not compare favorably with my hand work of fifteen and even twenty years, standing—this, of course, in good solid tooth structure—while the mallet work of *five* years' trial, in softer teeth, does not compare favorably with that of the same length of time, and even eight years done by gentle, steady pressure, and with soft foil.

Added to this I have noticed that in a large proportion of the gold *successes* which have been brought to my notice, the patients have each remarked that they remembered thinking that their "head was going to be *pushed* off," while in a large proportion of *failures*, the remark has been depreciatingly made that it "ought to have lasted, for it was *pounded* in hard enough." I have drawn, what I thought, a natural inference.

Accepting, then, the view that much waste of time was concommitant with less serviceable results, I feel it my duty, on the score of tooth salvation, to urge upon the younger and less experienced of my professional brethren an indulgence in a large share of hand pressure soft foil, cylinder, pellet rope and ribbon work, at least until *time* the great decider, shall have told each what he could *certainly* expect from varied effort.

I recognize as fully as any one the beautiful results which enthusiastic experts with the mallet are constantly producing—but these are mostly made in cases where *my convictions* to the use of *gold* at all; while I frequently witness the *hours* of clinics expended in malleting cohesive gold into articulating cavities of bicuspids and molars, I am sure, *from experience*, a few pellets of soft foil could be hand-pressed, in one quarter of the time, into a filling, which, I think, would prove really the more durable.—*St. Louis Dental Quart.rly.*

